# Safety and efficacy of a modified XELOX adjuvant regimen for patients with operated stage III colon cancer: a Chinese single-center experience

**DOI:** 10.1186/s40880-019-0400-x

**Published:** 2019-10-16

**Authors:** Jianhong Peng, Weihao Li, Rongxin Zhang, Junzhong Lin, Jinghua Tang, Yongshan Wen, Zhenhai Lu, Xiaojun Wu, Zhizhong Pan

**Affiliations:** Department of Colorectal Surgery, State Key Laboratory of Oncology in South China, Collaborative Innovation Center for Cancer Medicine, Sun Yat-sen University Cancer Center, 651 Dongfeng Road East, Guangzhou, Guangdong 510060 P. R. China

**Keywords:** Colon cancer, Adjuvant chemotherapy, Oxaliplatin, Capecitabine, XELOX, Efficacy, Safety

## Abstract

**Background:**

A fixed 8-cycle oxaliplatin and capecitabine (XELOX) regimen has been the standard adjuvant therapy for patients with stage III colon cancer. However, completing the full-cycle of oxaliplatin is often associated with severe neurotoxicity. To spare patients from the toxic effects, without comprising the required efficacy, we evaluated the safety and efficacy of a modified XELOX (mXELOX) adjuvant chemotherapy regimen with 6 cycles of oxaliplatin and a full cycle of capecitabine.

**Methods:**

We retrospectively analyzed 330 eligible patients with stage III colon cancer who underwent curative tumor resection followed by mXELOX, standard XELOX or unfinished XELOX adjuvant chemotherapy between December 2007 and April 2015. Associated prognostic factors were investigated and their disease-free survival (DFS) and overall survival (OS) rates were also determined and compared among the different regimen groups.

**Results:**

Compared with the standard XELOX group, the mXELOX group had lower total incidence rates of neurotoxicity (39.3% *vs.* 76.2%, *P* < 0.001), leucopenia (53.6% *vs.* 69.8%, *P* = 0.017) and thrombocytopenia (38.1% *vs.* 56.3%, *P* = 0.011). The standard XELOX and mXELOX adjuvant chemotherapy regimens presented with comparable 3-year DFS rates (86.3% *vs.* 89.2%; *P* = 0.838) and 3-year OS rates (92.7% *vs.* 97.6%; *P *= 0.227). Compared to unfinished XELOX chemotherapy, the oncologic benefits of the mXELOX regimen were greater for patients with T4 tumors (3-year DFS: Hazard ratio [HR], 2.184; 95% confidence interval [CI], 1.051–4.540; *P* = 0.036; 3-year OS: HR, 4.529; 95% CI 1.245–16.479; *P* = 0.022) and for high-risk patients (3-year DFS: HR, 1.962; 95% CI 0.964–3.993; *P *= 0.044; 3-year OS: HR, 4.193; 95% CI 1.182–14.874; *P* = 0.026).

**Conclusions:**

The mXELOX adjuvant chemotherapy presented a comparable survival benefit and lower incidence of toxicity than standard XELOX chemotherapy. It could be an alternative treatment for high-risk patients with operated stage III colon cancer.

## Background

Colorectal cancer (CRC) is one of the most common cancers and a leading cause of cancer death worldwide [1]. Especially in China, it is an increasingly important obstacle to the gains in life expectancy [2, 3]. Approximately one-third of patients with colon cancer have regional lymph node involvement; stage III disease at diagnosis [3, 4]. Curative surgical resection followed by adjuvant chemotherapy has been well-established and widely proposed as a standard clinical practice for patients with resected stage III colon cancer [5, 6]. Since the publication of the results of the Multicenter International Study of Oxaliplatin/5-fluorouracil/Leucovorin in the Adjuvant Treatment of Colon Cancer (MOSAIC) trial confirmed that adjuvant treatment with oxaliplatin plus 5-fluorouracil could improve the 5-year disease-free survival (DFS) rate and 6-year overall survival (OS) rate of stage III colon cancer patients by 7.5% and 4.2%, respectively [7, 8], oxaliplatin-based chemotherapy has been recommended as the standard postoperative treatment for these patients. Moreover, previous guidelines have suggested that the administration of a fixed 6-month oxaliplatin-based adjuvant chemotherapy for all the patients with stage III colon cancer, regardless of their risk stratifications [5, 9]. Therefore, adjuvant chemotherapy of stage III colon cancer was recognized as a unified treatment pattern.

In fact, not all patients benefit from oxaliplatin-containing adjuvant chemotherapy. As we previously reported, 20%–30% of these patients ultimately develop postoperative metastases [10, 11]. In addition, adjuvant treatment, especially with regimens containing oxaliplatin, is associated with considerable toxicity, especially chemotherapy-induced peripheral neuropathy. As a result, approximately 50% of patients fail to finish the planned therapy dose due to severe toxicity [12]. Therefore, an abbreviated duration of oxaliplatin-containing adjuvant therapy may be a feasible way to avoid or reduce the toxicities in some patients without impairing their oncologic outcomes. Recently, the International Duration Evaluation of Adjuvant Therapy (IDEA) trial compared the non-inferiority of stage III colon cancer patients receiving oxaliplatin-containing adjuvant therapy for 3 and 6 months [13]. Although the non-inferiority of 3-months treatment versus 6-months was not confirmed in the overall study population, the XELOX regimen showed more efficient in that the non-inferiority of the shorter duration was confirmed for XELOX but not for FOLFOX regimen. These findings revealed that the XELOX regimen may be a unique but not equivalent chemotherapy to FOLFOX for treating stage III colon cancer. Further analyses of IDEA trail indicated that the 3-month XELOX adjuvant chemotherapy appeared to be sufficient for low-risk patients. Accordingly, the most recent National Comprehensive Cancer Network (NCCN) guidelines recommend a 3 month XELOX adjuvant chemotherapy regimen for treating low-risk patients [14]. However the actual oncologic benefits gained from the 6-month XELOX adjuvant chemotherapy have not yet been conclusively established for high-risk patients.

Acceptable toxicities and compliance were observed in the patients with stage III colon cancer when administered eight courses of capecitabine with a dose of 1250 mg/m^2^ orally, twice daily, on days 1–14, every 21 days [15, 16]. Enlightened by the results of the IDEA trial, we hypothesized that a modified XELOX (mXELOX) adjuvant chemotherapy with 6 cycles of oxaliplatin and a full cycle of capecitabine might have comparable efficacy with acceptable toxicities compared with the 6-month standard XELOX adjuvant chemotherapy regimen for patients with stage III colon cancer, particularly in the high-risk subgroup. However, few studies have assessed the clinical efficacy of mXELOX adjuvant chemotherapy in patients with stage III colon cancer. Herein, the present study aimed to evaluate the survival benefit and safety of mXELOX adjuvant chemotherapy in operated stage III colon cancer patients and to further identify the subgroup that may potentially benefit from mXELOX adjuvant chemotherapy.

## Patients and methods

### Patient selection

The medical records of 450 consecutive patients were reviewed. All patients were diagnosed with stage III colon cancer and underwent tumor resection followed by adjuvant chemotherapy between November 2007 and April 2015 at Sun Yat-sen University Cancer Center (Guangzhou, China). All cases were staged according to the 8th edition American Joint Committee on Cancer (AJCC) staging system. The inclusion criteria were as follows: (1) histologically confirmed colorectal adenocarcinoma; (2) underwent curative resection of colon tumor; (3) received adjuvant chemotherapy with XELOX regimen (oxaliplatin 130 mg/m^2^ administered intravenously on day 1 and capecitabine 1000 mg/m^2^ administered orally twice daily on days 1–14 for a 3-week cycle); (4) had complete record of the whole treatment; (5) did not received anticancer therapy before tumor resection; and (6) underwent at least a 6-month follow-up after the delivery of the first cycle chemotherapy. The patient demographics, tumor characteristics, and adjuvant chemotherapy cycles were carefully reviewed. The present study was performed according to the ethical standards of the World Medical Association Declaration of Helsinki and was approved by the Institutional Review Board and Independent Ethics Committees of Sun Yat-sen University Cancer Center. The informed consent requirement was waived based on the nature of this retrospective study, in which patient data were kept confidential.

### Definition and measurements

The recommended XELOX adjuvant chemotherapy started 3–6 weeks after curative surgery. If a patient could not tolerate the full dose or suffered from severe toxic effects, the chemotherapy was stopped. According to the different cycles of the XELOX adjuvant chemotherapy performed, patients were divided into the modified, standard, and unfinished XELOX subgroups. mXELOX was defined as 6 cycles of the XELOX regimen plus 2 subsequent cycles of capecitabine alone, which consisted 6-cycle of oxaliplatin and 8-cycle of capecitabine. Standard XELOX referred to the XELOX regimen which completed the entire 8 cycles (8-cycle of oxaliplatin and 8-cycle of capecitabine). Unfinished XELOX was the adjuvant chemotherapy with no more than 6 cycles of XELOX, which consists of 6 or fewer cycles of oxaliplatin or capecitabine use. Some patients failed to finish the complete the planned cycle or the treatment mainly contributing to the severe toxicity of the adjuvant chemotherapy or poor compliance to the subsequent cycle of therapy. Right-sided colon cancer included the cecum, ascending colon, hepatic flexure, and transverse colon cancer, whereas left-sided colon cancer included the splenic flexure, descending colon, and sigmoid colon cancer. Pathological assessments and staging of the resected specimens were confirmed according to tumor-node-metastasis (TNM) classification by two independent pathologists. Patients with a combination of T1–3 and N1 disease were classified into the low-risk group, while patients with T4 or N2 disease were classified into the high-risk group. The intensity of the adverse events during chemotherapy was graded according to the National Cancer Institute Common Terminology Criteria for Adverse Events (NCI CTCAE), version 4.0. A complete laboratory assessment was performed before each treatment cycle (2 or 3 days before each cycle).

### Follow-up

The primary endpoint was DFS, and the secondary endpoint was OS. DFS was defined as the interval from surgery to disease recurrence, death, or the last follow-up. OS was defined as the interval from the date of surgery until death of any cause or the last follow-up. Patients without any event (metastasis or death) at the last follow-up date were regarded as random censoring. All patients were observed through subsequent visits every 3 months for 2 years and then semiannually until 3 years after surgery. Physical examination, blood tests of carcinoembryonic antigen (CEA) and carbohydrate antigen 19-9 (CA19-9) levels, abdominal ultrasonography, and chest X-ray were conducted every 3 months postoperatively. Chest/abdominal/pelvic computed tomography (CT) and colonoscopy were performed annually. If abnormality in CEA or CT were found, a abdominal/pelvic magnetic resonance imaging (MRI) or positron emission tomography/computed tomography (PET-CT) would be performed for further tumor detection. The last follow-up visit was in April 2018.

### Statistical analysis

All statistical analyses were performed using IBM SPSS statistics software, version 21.0 (IBM Corp., Armonk, NY, USA). Categorical variables are given as percentages and were compared using the Chi square or Fisher’s exact test when appropriate. The OS and DFS rates were estimated with the Kaplan–Meier method, and the differences between groups were then assessed with the log-rank test. Parameters for which *P* < 0.05 in the univariate Cox models were further assessed in multivariate Cox models. Hazard ratios (HRs) and 95% confidence intervals (CIs) were subsequently calculated. All of the statistical tests were two-sided. *P* < 0.05 was considered significant.

## Results

### Patient characteristics

Among the 450 patients, 120 patients were excluded for the following reasons: treatment with other regimens of adjuvant chemotherapy (*n* = 91), follow-up < 3 months (*n* = 11) and incomplete tumor resection (*n* = 18). Overall, 330 eligible patients were identified for analysis in the present study. Their demographic features and clinicopathological characteristics are summarized in Table 1. Of the 330 patients, 189 (57.3%) were males, and 141 (42.7%) were females, with a median age of 55 years (range, 19–85 years). Regarding tumor location, 131 (39.7%) patients presented with right-sided colon cancer, whereas 199 (60.3%) patients had left-sided colon cancer. The median number of resected lymph nodes was 15 (range, 2–63). With respect to risk stratification, 131 (39.7%) patients were identified as low-risk patients, while 199 (60.3%) patients were diagnosed as high-risk patients. In total, 5 (1.5%) patients experienced postoperative complications, including 3 (0.9%) with intestinal obstruction, 1 (0.3%) with anastomotic leakage, and 1 (0.3%) with incision infection. Among the 330 investigated patients, 84 (25.5%) received mXELOX adjuvant chemotherapy, 126 (38.2%) received standard XELOX adjuvant chemotherapy, and 120 (36.4%) received unfinished XELOX adjuvant chemotherapy. Among the 120 patients who received unfinished XELOX adjuvant chemotherapy, 14 (11.7%) had 1 cycle, 16 (13.3%) had 2 cycles, 11 (9.2%) had 3 cycles, 15 (12.5%) had 4 cycles, 15 (12.5%) had 5 cycles, and 49 (40.8%) had 6 cycles of XELOX adjuvant chemotherapy.

**Table 1:** Demographic and clinicopathological variables of the 330 investigated stage III colon cancer patients

Variables	All patients [cases (%)]
Age [median (range), years]	55 (19–85)
Gender	
Male	189 (57.3)
Female	141 (42.7)
BMI [median (range), kg/m^2^]	22.3 (14.3–34.2)
Tumor size [median (range), cm]	4 (0.8–15)
Tumor location	
Cecum	15 (4.5)
Ascending colon	55 (16.7)
Hepatic flexure	30 (9.1)
Transverse colon	31 (9.4)
Splenic flexure	6 (1.8)
Descending colon	31 (9.4)
Sigmoid colon	162 (49.1)
Tumor differentiation	
Well/moderately differentiated	238 (72.1)
Poor/undifferentiated	92 (27.9)
T stage	
T1–2	16 (4.8)
T3	156 (47.3)
T4	158 (47.9)
No. of resected lymph nodes [median (range)]	15 (2–63)
N stage	
N1	233 (70.6)
N2	97 (29.4)
Preoperative serum CEA (ng/mL)	
≤ 5	199 (60.3)
> 5	131 (39.7)
Preoperative serum CA19-9 (U/mL)	
≤ 24	228 (69.1)
> 24	102 (30.9)
Risk stratification	
Low risk	131 (39.7)
High risk	199 (60.3)

*BMI* body mass index, *CEA* carcinoembryonic antigen, *CA19-9* carbohydrate antigen 19-9

### Clinicopathological features of patients treated with different XELOX chemotherapy regimens

As shown in Table 2, a higher T4 proportion was more common in the mXELOX group than that in the standard XELOX group (71.4% *vs.* 37.3%; *P* < 0.001) and unfinished XELOX group (71.4% *vs.* 42.5%; *P *< 0.001). Patients in the mXELOX group were more likely to be stratified as high-risk patients than those in the standard XELOX group (75.0% *vs.* 54.0%; *P *= 0.002) and unfinished XELOX group (75.0% *vs.* 56.7%; *P *= 0.008). There were no significant differences observed regarding age, gender, body mass index (BMI), tumor size, tumor location, tumor differentiation, N stage, number of resected lymph nodes, preoperative serum CEA level, or preoperative serum CA19-9 level.

**Table 2:** Demographic and clinicopathological variables of 330 patients with operated stage III colon cancer in the standard, modified and unfinished XELOX group

Variables	Standard XELOX (*n *= 126, %)	Modified XELOX (*n *= 84, %)	Unfinished XELOX (*n *= 120, %)	*P* value 1	*P* value 2
Age (years)				0.762	0.454
≤ 60	85 (67.5)	59 (70.2)	78 (65.0)		
> 60	41 (32.5)	25 (29.8)	42 (35.0)		
Gender				1.000	0.386
Male	69 (54.8)	46 (54.8)	74 (61.7)		
Female	57 (45.2)	38 (45.2)	46 (38.3)		
BMI (kg/m^2^)				0.371	0.439
< 18.5	12 (9.5)	6 (7.1)	14 (11.7)		
18.5–25.0	83 (65.9)	63 (75.0)	81 (67.5)		
> 25.0	31 (24.6)	15 (17.9)	25 (20.8)		
Tumor size (cm)				0.945	0.885
≤ 4	77 (61.1)	52 (61.9)	73 (60.8)		
> 4	49 (38.9)	32 (38.1)	47 (39.2)		
Tumor location				0.382	0.567
Right-sided colon	43 (34.1)	34 (40.5)	54 (45.0)		
Left-sided colon	83 (65.9)	50 (59.5)	66 (55.0)		
Tumor differentiation				0.751	0.536
Well/moderately differentiated	91 (72.2)	63 (75.0)	84 (70.0)		
Poor/undifferentiated	35 (27.8)	21 (25.0)	36 (30.0)		
T stage				< *0.001*	< *0.001*
T1–2	7 (5.6)	1 (1.2)	8 (6.7)		
T3	72 (57.1)	23 (27.4)	61 (50.8)		
T4	47 (37.3)	60 (71.4)	51 (42.5)		
Numbers of resected lymph nodes				0.145	0.433
< 12	27 (21.4)	26 (31.0)	31 (25.8)		
≥ 12	99 (78.6)	58 (69.0)	89 (74.2)		
N stage				0.541	0.438
N1	90 (71.4)	56 (66.7)	87 (72.5)		
N2	36 (28.6)	28 (33.3)	33 (27.5)		
Preoperative serum CEA (ng/mL)				0.063	0.657
≤ 5	67 (53.2)	56 (66.7)	76 (63.3)		
> 5	59 (46.8)	28 (33.3)	44 (36.7)		
Preoperative serum CA19-9 (U/mL)				0.275	0.092
≤ 24	86 (68.3)	64 (76.2)	78 (65.0)		
> 24	40 (31.7)	20 (23.8)	42 (35.0)		
Risk stratification				*0.002*	*0.008*
Low risk	58 (46.0)	21 (25.0)	52 (43.3)		
High risk	68 (54.0)	63 (75.0)	68 (56.7)		

*XELOX* oxaliplatin and capecitabine regimen, *BMI* body mass index, *CEA* carcinoembryonic antigen, *CA19-9* carbohydrate antigen 19-9

Right-sided colon cancer included the cecum, ascending colon, hepatic flexure, and transverse colon cancer, whereas left-sided colon cancer included the splenic flexure, descending colon, and sigmoid colon cancer. *P* value 1 is the result of comparing the standard XELOX regimen group with the modified XELOX regimen group. *P* value 2 is the result of comparing the unfinished XELOX regimen with the modified XELOX regimen group

### Adverse events

The major adverse events during adjuvant chemotherapy with mXELOX and standard XELOX are presented in Table 3. No patients suffering from grade 3/4 adverse event were hospitalized for treatment. Compared with the mXELOX group, the standard XELOX group showed a higher total incidence of neurotoxicity (76.2% *vs.* 39.3%, *P* < 0.001), especially grade 1 (48.4% *vs.* 23.8%, *P* < 0.001) and grade 2 neurotoxicity (24.6% *vs.* 13.1%, *P* = 0.041). Additionally, the standard XELOX group had a higher total occurrence rate of leucopenia (69.8% *vs.* 53.6%, *P* = 0.017) and thrombocytopenia (56.3% *vs.* 38.1%, *P *= 0.011) than the mXELOX group. However, there were no significant differences in the occurrence rates of nausea and vomiting, diarrhea, hand-foot syndrome, and hepatic disorder between the two groups. The major adverse events in the unfinished XELOX group were neurotoxicity (*n *= 48, 40.0%) and leucopenia (*n *= 47, 39.1%).

**Table 3:** Comparison of treatment-related adverse events between the modified XELOX and standard XELOX groups

Adverse events	standard XELOX (*n *= 126, %)	modified XELOX (*n *= 84, %)	*P* value
Neurotoxicity			
Total	96 (76.2)	33 (39.3)	*< 0.001*
Grade 1	61 (48.4)	20 (23.8)	*< 0.001*
Grade 2	31 (24.6)	11 (13.1)	*0.041*
Grade 3–4	4 (3.2)	2 (2.4)	0.311
Leucopenia			
Total	88 (69.8)	45 (53.6)	*0.017*
Grade 1	56 (44.4)	29 (34.5)	0.196
Grade 2	22 (17.5)	10 (11.9)	0.329
Grade 3–4	10 (7.9)	6 (7.1)	0.832
Thrombocytopenia			
Total	75 (59.5)	32 (38.1)	*0.011*
Grade 1	45 (35.7)	17 (20.2)	*0.016*
Grade 2	26 (20.6)	12 (14.3)	0.242
Grade 3–4	4 (3.2)	3 (3.6)	0.592
Nausea/vomiting			
Total	46 (36.5)	26 (31.0)	0.861
Grade 1	27 (21.4)	13 (15.5)	0.221
Grade 2	18 (14.3)	10 (11.9)	0.683
Grade 3–4	1 (0.8)	3 (3.6)	0.149
Diarrhea			
Total	17 (13.5)	11 (13.1)	0.934
Grade 1	9 (7.1)	7 (8.3)	0.794
Grade 2	5 (4.0)	3 (3.6)	0.883
Grade 3–4	3 (2.4)	1 (1.2)	0.536
Hand-foot syndrome			
Total	50 (39.7)	23 (27.4)	0.067
Grade 1	32 (25.4)	15 (17.9)	0.238
Grade 2	13 (10.3)	5 (6.0)	0.128
Grade 3–4	5 (4.0)	3 (3.6)	0.251
Hepatic disorder			
Total	46 (36.5)	22 (26.2)	0.134
Grade 1	34 (27.0)	17 (20.2)	0.325
Grade 2	10 (7.9)	4 (4.8)	0.414
Grade 3–4	2 (1.6)	1 (1.2)	0.817

*XELOX* oxaliplatin and capecitabine regimen

The listed grades of peripheral sensory neurotoxicity represent the maximal levels at any time

### Survival analysis

**Fig. 1:**
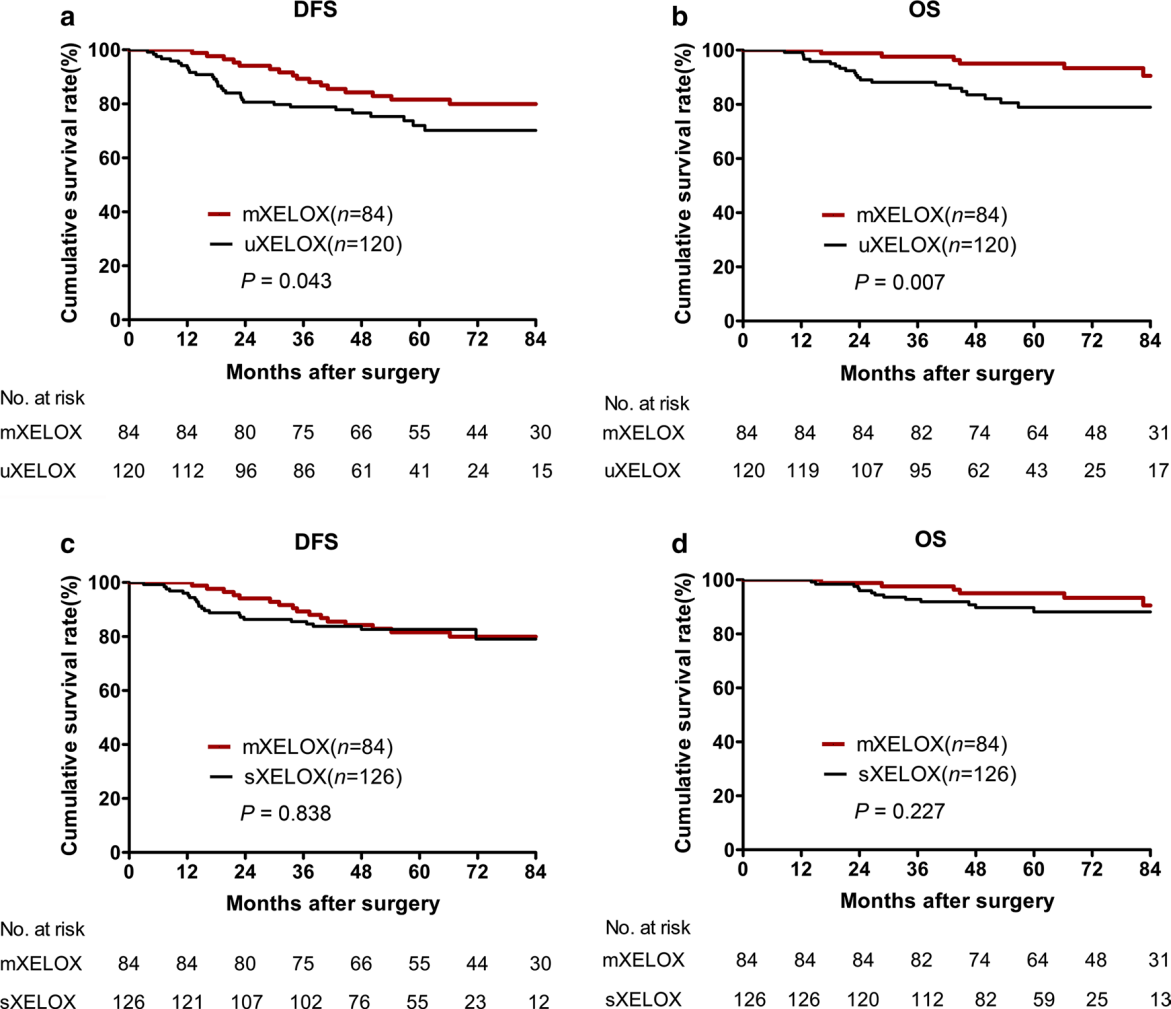
Kaplan-Meier curves of patients with stage III colon cancer grouped by modified XELOX, standard XELOX, and unfinished XELOX regimens. **a** Comparison of disease-free survival between the modified XELOX group and the unfinished XELOX group. **b** Comparison of overall survival between the modified XELOX group and the unfinished XELOX group. **c** Comparison of disease-free survival between the modified XELOX group and the standard XELOX group. **d** Comparison of overall survival between the modified XELOX group and the standard XELOX group. Abbreviations: XELOX, oxaliplatin and capecitabine regimen. DSF, disease-free survival; OS, overall survival

The median follow-up period for all patients was 60 months (range, 8-115 months). Seventy-two (21.8%) patients experienced tumor metastasis, while 40 (12.1%) patients ultimately died of tumor progression. The 3-year DFS and OS rates for the entire study population were 84.0% and 92.3%. As shown in Table 4, the total postoperative metastasis rate was significantly higher in the unfinished XELOX group than in the mXELOX group (29.2% *vs.* 16.7%, *P* = 0.046). In addition, abdominopelvic metastasis was more common in the unfinished XELOX group than in the mXELOX group (11.7% *vs.* 2.4%, *P* = 0.017). However, the incidence of total postoperative metastasis, liver metastasis, lung metastasis, and abdominopelvic metastasis were comparable between the standard XELOX group and the mXELOX group. The 3-year DFS and OS rates were significantly lower in the unfinished XELOX group than in the mXELOX group (DFS, 78.9% *vs.* 89.2%, *P* = 0.043, Fig. 1a; OS, 88.2% *vs.* 97.6%, *P* = 0.007, Fig. 1b). There were no significant differences in the 3-year DFS or OS rates between the standard XELOX and mXELOX groups (DFS, 86.3% *vs.* 89.2%, *P* = 0.838, Fig. 1c; OS, 92.7% *vs.* 97.6%, *P* = 0.227, Fig. 1d).

**Table 4:** Postoperative metastatic patterns of patients with stage III colon cancer after curative treatment

Metastatic parameters	Standard XELOX (*n *= 126, %)	Modified XELOX (*n *= 84, %)	Unfinished XELOX (*n *= 120, %)	*P* value 1	*P* value 2
Postoperative metastasis				0.861	*0.046*
Present	23 (18.3)	14 (16.7)	35 (29.2)		
Absent	103 (81.7)	70 (83.3)	85 (70.8)		
Liver metastasis				0.821	0.798
Present	8 (6.3)	6 (7.1)	11 (9.2)		
Absent	118 (93.7)	78 (92.9)	109 (90.8)		
Lung metastasis				0.570	0.739
Present	9 (7.1)	4 (4.8)	7 (5.8)		
Absent	117 (92.9)	80 (95.2)	113 (94.2)		
Abdominopelvic metastasis				0.735	*0.017*
Present	4 (3.2)	2 (2.4)	14 (11.7)		
Absent	122 (96.8)	82 (97.6)	106 (88.3)		

*XELOX* oxaliplatin and capecitabine regimen

*P* value 1 is the result of comparing the standard XELOX regimen group with the modified XELOX regimen group. *P* value 2 is the result of comparing the unfinished XELOX regimen with the modified XELOX regimen group

### Prognostic analysis of clinical factors

**Fig. 2:**
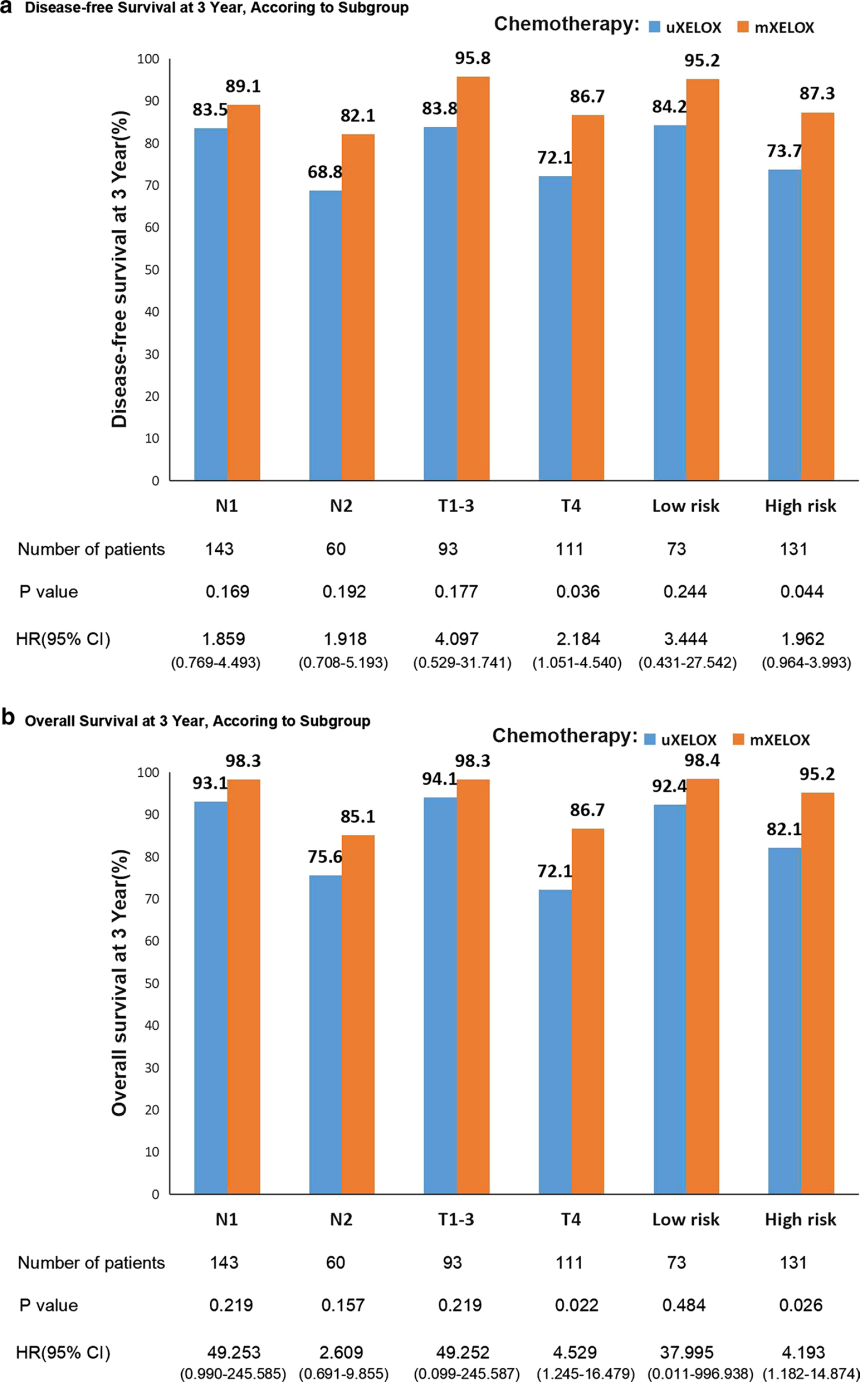
Subgroup analysis of the 3-year survival rate of patients with stage III colon cancer grouped by modified XELOX and unfinished XELOX according to T stage, N stage, and risk stratification. **a** Subgroup analysis of the 3-year disease-free survival rate. **b** Subgroup analysis of the 3-year overall survival rate. *XELOX* oxaliplatin and capecitabine regimen, *HR* hazard ratio, *CI* confidence interval

As shown in Table 5, univariate analysis revealed that age more than 60 years (HR, 1.702; 95% CI 1.057–2.702; *P* = 0.026), male sex (HR, 1.891; 95% CI 1.132–3.159; *P* = 0.015), high preoperative CEA level (HR, 1.721; 95% CI 1.072–2.760; *P* = 0.024), high preoperative CA19-9 level (HR, 2.674; 95% CI 1.667–4.288; *P *< 0.001) and unfinished XELOX regimens (HR, 1.668; 95% CI 1.010–3.057; *P* = 0.043) were significant risk factors for 3-year DFS. In addition, T4 tumor, high-risk stratification, unfinished XELOX regimen, and high preoperative CA19-9 level were significant risk factors for the 3-year OS. In multivariable analysis, male sex (HR, 2.322; 95% CI 1.201–4.492; *P* = 0.012) and high preoperative CA19-9 level (HR, 3.089; 95% CI 1.654–5.770; *P* < 0.001) were identified as independent negative predictors of 3-year DFS, whereas unfinished XELOX adjuvant chemotherapy (HR, 3.869; 95% CI 1.515–9.880; *P* = 0.005) was identified as an independent negative predictor of 3-year OS (Table 6). Comparisons of the 3-year DFS and OS rates between the mXELOX and unfinished XELOX groups stratified by T stage, N stage and risk stratification are shown in Fig. 2. The oncologic benefits of the mXELOX regimen were especially noticeable for patients with T4 tumors (3-year DFS: HR, 2.184; 95% CI 1.051–4.540; *P *= 0.036; Fig. 2a; 3-year OS: HR, 4.529; 95% CI 1.245–16.479; *P *= 0.022; Fig. 2b) and for high-risk patients (3-year DFS: HR, 1.962; 95% CI 0.964–3.993; *P* = 0.044; Fig. 2a; 3-year OS: HR, 4.193; 95% CI 1.182–14.874; *P* = 0.026; Fig. 2b).

**Table 5:** Univariate analyses of prognostic factors for disease-free survival and overall survival in patients with stage III colon cancer who received curative treatment

Variables	Disease-free survival		Overall survival	
	HR (95% CI)	*P* value	HR (95% CI)	*P* value
Age, year (> 60 *vs.* ≤ 60))	*1.702* *(1.057–2.702)*	*0.026*	1.465 (0.778–2.758)	0.237
Gender (male *vs.* female)	*1.891* *(1.132–3.159)*	*0.015*	1.877 (0.954–3.693)	0.068
BMI, kg/m^2^ (< 18.5 *vs.* ≥ 18.5)	1.725 (0.538–5.527)	0.036	0.775 (0.274–2.191)	0.630
Tumor size, cm (> 4 *vs.* ≤ 4)	0.781 (0.483–1.264)	0.314	0.962 (0.516–1.794)	0.902
Tumor location (right-sided colon *vs.* left-sided colon)	0.798 (0.496–1.285)	0.353	0.400 (0.213–1.754)	0.557
Differentiation (poor *vs.* well/moderately differentiated)	1.354 (0.815–2.248)	0.242	1.245 (0.632–2.451)	0.527
T stage (T4 *vs.* T1–3)	1.446 (0.895–2.337)	0.132	*2.311* *(1.170–4.564)*	*0.016*
Numbers of resected lymph nodes (< 12 *vs.* ≥ 12)	1.356 (0.817–2.250)	0.239	0.938 (0.458–1.919)	0.860
N stage (N2 *vs.* N1)	1.334 (0.813–2.190)	0.225	1.817 (0.971–3.403)	0.062
Preoperative serum CEA, ng/mL (> 5 *vs.* ≤ 5)	*1.721* *(1.072–2.760)*	*0.024*	1.104 (0.590–2.067)	0.757
Preoperative serum CA19-9, U/mL (> 24 *vs.* ≤ 24)	*2.674* *(1.667–4.288)*	*< 0.001*	*2.216* *(1.190–4.124)*	*0.012*
Risk stratification (high *vs.* l ow)	1.262 (0.764–2.086)	0.363	*2.441* *(1.122–5.313)*	*0.024*
Adjuvant chemotherapy (unfinished XELOX *vs.* modified XELOX)	*1.668* *(1.010–3.057)*	*0.043*	*3.256* *(1.305–8.126)*	*0.011*
Adjuvant chemotherapy (standard XELOX *vs.* modified XELOX)	0.935 (0.489–1.785)	0.838	0.551 (0.207–1.468)	0.233

*XELOX* oxaliplatin, and capecitabine regimen, *BMI* body mass index, *CEA* carcinoembryonic antigen, *CA19-9* carbohydrate antigen 19-9, *HR* hazard ratio, *CI* confidence interval

Right-sided colon cancer included the cecum, ascending colon, hepatic flexure, and transverse colon cancer, whereas left-sided colon cancer included the splenic flexure, descending colon, and sigmoid colon cancer

**Table 6:** Multivariate analyses of prognostic factors for disease-free survival and overall survival in patients with stage III colon cancer who received curative treatment

Variables	Disease-free survival		Variables	Overall survival	
	HR (95% CI)	*P* value		HR (95% CI)	*P* value
Age, year (> 60 *vs.* ≤ 60)	1.787 (0.995–3.209)	0.052	T stage (T4 *vs.* T1–3)	1.602 (0.465–5.520)	0.455
Gender (male *vs.* female)	*2.322* *(1.201–4.492)*	*0.012*	Risk stratification (high *vs.* low)	3.259 (0.654–6.236)	0.149
Adjuvant chemotherapy (unfinished XELOX *vs.* modified XELOX)	1.448 (0.787–2.664)	0.235	Adjuvant chemotherapy (unfinished XELOX *vs.* modified XELOX)	*3.869* *(1.515–9.880)*	*0.005*
Preoperative serum CA19-9, U/mL (> 24 *vs.* ≤ 24)	*3.089* *(1.654–5.770)*	*< 0.001*	Preoperative serum CA19-9, U/mL (> 24 *vs.* ≤ 24)	2.037 (0.946–4.387)	0.069
Preoperative serum CEA, ng/mL (> 5 *vs.* ≤ 5)	1.147 (0.617–2.132)	0.666			

*XELOX* oxaliplatin and capecitabine regimen, *CEA* carcinoembryonic antigen, *CA19-9* carbohydrate antigen 19-9, *HR* hazard ratio, *CI* confidence interval

## Discussion

In this retrospective study, we investigated the effects of mXELOX adjuvant chemotherapy on prognostic efficacy and safety by comparing standard XELOX and unfinished XELOX adjuvant chemotherapy regimens among operated stage III colon cancer patients. Herein, we found that the 3-year DFS and OS rates were better in the mXELOX group than those in the unfinished XELOX group. As expected, the 3-year survival rate of the mXELOX group was comparable to that of the standard XELOX group, with acceptable safety. The results provided the first evidence supporting the administration of the mXELOX adjuvant chemotherapy regimen for stage III colon cancer patients.

The benefit of oxaliplatin-containing adjuvant chemotherapy has been clearly proven for patients with stage III colon cancer and may eradicate micrometastases after surgical resection, reducing the likelihood of disease recurrence and potentially increasing the curative rates postoperatively [17]. However, a long duration of oxaliplatin treatment causes cumulative toxic effects, especially neurotoxicity, which is the single main dose-limiting factor in the treatment of colorectal cancer [18, 19]. According to the MOSAIC trial, the incidence of grade 3 acute peripheral sensory neurotoxicity among oxaliplatin-treated patients was 12%, and a similar proportion of patients developed chronic peripheral neurotoxicity that unpredictably may last for years [20]. Such toxic effects can potentially affect patients’ activities of daily living for the rest of their lives [21]. The IDEA study showed that a shorter duration of oxaliplatin-based adjuvant therapy resulted in a significantly lower incidence and severity of adverse events, including neurotoxicity, hand-foot syndrome, mucositis, nausea, fatigue, and diarrhea [13]. Similar to that of the IDEA study, the data of our study showed that a 2-cycle shorter duration of oxaliplatin treatment in the mXELOX regimen presented a significantly lower incidence and severity of adverse events, especially neurotoxicity but also hematological side effects, such as leucopenia and thrombocytopenia. We considered that a shorter duration of oxaliplatin-based chemotherapy resulted in the advantages of safety control and life quality improvement.

Survival data from this study revealed that no more than 6 cycles of adjuvant chemotherapy presented a higher rate of total postoperative metastasis, resulting in a worse 3-year DFS and OS than those undergoing the 8 cycles of adjuvant chemotherapy. In addition, unfinished XELOX was identified as an independent negative risk factor for 3-year OS. Similarly, population-based studies have confirmed that compared with the early termination of adjuvant therapy, the completion of planned adjuvant therapy is associated with lower overall and colon cancer-specific mortality rates, a 45% reduction in the risk of recurrence and a 51% reduction in mortality [22, 23]. In the current study, the cut-off for oxaliplatin-based treatment completion was set at 6 cycles; accordingly, we considered addition of 2 cycle of capecitabine or XELOX regimen to 6 cycles of oxaliplatin-based adjuvant chemotherapy was necessary for disease control.

On the other hand, we scheduled mXELOX chemotherapy with 8 cycles of capecitabine to complement the full cycle of adjuvant chemotherapy under lower subsequent toxicities. To our interest, mXELOX chemotherapy obtained comparable 3-year DFS and OS rates to those following a standard full XELOX chemotherapy regimen, and the mXELOX regimen showed a superior survival outcome than did the unfinished XELOX regimen. This result is in line with that from a previous study showing that patients treated with capecitabine monotherapy who completed 6–8 cycles of capecitabine monotherapy had better cancer-specific survival than did those who received 1–5 cycles of the treatment [24]. In the current study, approximately two-thirds of patients received 8 cycles of capecitabine with a low incidence (4%) of grade 3–4 hand-foot syndrome. An observational study of adjuvant therapy with capecitabine in patients with colon cancer also reported that six or more cycles of treatment were completed by 77.9% of patients [25]. Oral capecitabine is well tolerated by the patients, and the high rate of compliance to treatment may be attributed to the completion of drug delivery. Accordingly, we recommend the completion of oral capecitabine, which can confer to improve survival benefit with controllable toxicities, in patients with stage III colon cancer.

Our exploratory analysis further indicated that mXELOX adjuvant chemotherapy was more beneficial in the high-risk group. In general, patients with advanced disease were more likely to suffer from aggressive tumors, a high tumor burden, and aggravated immunosuppression [9, 26]. In this condition, the long treatment and full cycle of chemotherapy might be appropriate for high-risk patients. For the low-risk group, 3-month duration of XELOX adjuvant chemotherapy was sufficient due to the noninferiority of DFS compared with the DFS following 6-month adjuvant chemotherapy [13]. Therefore, our data also warrant a division of stage III colon cancer patients into low-risk and high-risk groups to reduce overtreatment and allow more individual treatment for stage III colon cancer.

Several limitations should be acknowledged in the present study. First, this retrospective study included an uncontrolled methodology and a limited number of patients from a single cohort. Although our study initially indicated the potential clinical efficacy of mXELOX adjuvant chemotherapy, the findings need to be validated in a prospective, multicenter clinical trial with a large population in the future. Second, the short follow-up duration was insufficient for 50.9% patients to evaluate 5-year survival outcomes, which may have led to an misestimation of the effect of mXELOX adjuvant chemotherapy on OS. In the current study, the identification of risk stratification depended only on the TNM stage, which may not represent an optimal prognostic tool for tailoring adjuvant treatment in a comprehensive transversal approach. Additionally, tumor molecular markers, such as microsatellite status, CpG island methylator phenotype (CIMP) status, BRAF mutations, and KRAS mutations as well as tumor immune infiltration have been linked to different recurrence risks and survival outcomes in patients with stage III colon cancer [27, 28]. Thus, it is necessary to include pathological, immunological and molecular prognostic markers for risk stratification in further studies.

## Conclusion

Compared to the standard 8-cycle XELOX chemotherapy, mXELOX adjuvant chemotherapy presented a comparable survival benefit and lower incidence rates of neurotoxicity and hematological toxicity. Moreover, mXELOX had a superior 3-year survival outcome than unfinished XELOX adjuvant chemotherapy, especially in high-risk patients. These data suggest that mXELOX adjuvant chemotherapy could serve as an alternative treatment for high-risk patients with operated stage III colon cancer.

## Data Availability

The datasets used and analyzed during the current study are available from the corresponding author on reasonable request. The authenticity of this article has been validated by uploading the key raw data onto the Research Data Deposit public platform (http://www.researchdata.org.cn), with the Approval Number as RDDA2019001192.

## Abbreviations

XELOX: oxaliplatin and capecitabine regimen
mXELOX: modified XELOX
DFS: disease-free survival
OS: overall survival
HR: hazard ratio
CI: confidence interval
CRC: colorectal cancer
MOSAIC: Multicenter International Study of Oxaliplatin/5-fluorouracil/Leucovorin in the Adjuvant Treatment of Colon Cancer
IDEA: International Duration Evaluation of Adjuvant Therapy
NCCN: National Comprehensive Cancer Network
AJCC: American Joint Committee on Cancer
TNM: tumor-node-metastasis
NCI CTCAE: National Cancer Institute Common Terminology Criteria for Adverse Events
CEA: carcinoembryonic antigen
CA19-9: carbohydrate antigen 19-9
CT: computed tomography
BMI: body mass index
CIMP: CpG island methylator phenotype
